# Editorial: Glycans: Masters of immunity, from cancers to inflammatory disease

**DOI:** 10.3389/fimmu.2022.1002679

**Published:** 2022-08-17

**Authors:** Richard Beatson, Heinz Läubli, Oliver M.T. Pearce, Celso A. Reis

**Affiliations:** ^1^ Centre for Inflammation and Tissue Repair, Respiratory Medicine, University College London, London, United Kingdom; ^2^ Laboratory for Cancer Immunotherapy, Department of Biomedicine and Medical Oncology, University Hospitalheinz, Basel, Switzerland; ^3^ Laboratory for Cancer Immunotherapy, Department of Internal Medicine, University Hospitalheinz, Basel, Switzerland; ^4^ Barts Cancer Institute, Queen Mary University of London, London, United Kingdom; ^5^ Instituto de Investigação e Inovação em Saú de (i3s), Universidade do Porto, Porto, Portugal; ^6^ Instituto de Patologia e Imunologia Molecular da Universidade do Porto (IPATIMUP), Porto, Portugal; ^7^ Instituto de Ciê ncias Biomé dicas Abel Salazar (ICBAS), Universidade do Porto, Porto, Portugal; ^8^ Faculdade de Medicina da Universidade do Porto (FMUP), Porto, Portugal

**Keywords:** glycans, immunity, cancer, inflammatory disease, glycoimmunology

As editors of this Research Topic, it was our pleasure to review a wide range of fascinating articles and reviews within the field. In this editorial we summarize the main findings and perspectives detailed within each of the accepted articles.

Hugonnet et al. give an overview on the different functions sialyltransferases have in cancer progression and inhibition of anti-cancer immunity. Hypersialylation – a term used for a cancer-associated increase in intratumoral sialic acid content – has been described many years ago and can be significantly supported by sialyltransferases. However, only recent elucidation of various mechanisms promoting cancer progression including engagement of Siglec receptors, stabilization of receptors and influencing of antigen presentation have led to further investigations to target hypersialylation and sialyltransferases for cancer therapy.

Mucins are well studied carriers of these hypersialylated glycans, and as such, in a hypothesis article, Hitchcock et al. propose to use an antibody against the cancer-associated TAG-72 mucin protein to determine the extent of surgery for patients with colorectal cancer (CRC). Mucins play an important role in cancer progression and metastasis formation. The use of cancer-associated changes in mucins could therefore be a valuable diagnostic and therapeutic target.

Hypersialylated glycans are well-known to engage Siglecs, however there is much complexity beyond this statement. Van Houtum et al. summarize the current role of Siglec receptors in the tumor microenvironment. Interactions of Siglec receptors with sialic acid-containing ligands have recently moved into the focus of a broad interest as the first Siglec-targeting antibodies as well as sialic acid-reducing compounds have reached early clinical stages of development.

An improved understanding of the Siglec-Ligand interactions described above is likely to prove useful in the application of this axis in regulating immune responsiveness. The current tools being used to explore the Siglec-ligand interaction, and in particular the physiological ligands, is discussed within the mini review by Jiang et al. In this review the advantages and disadvantages of the current methodologies to identify relevant Siglec ligands is summarised, including affinity purification, proximity labelling, and genetic modifications of cells and genome-wide screening, with consideration given to the *cis* or *trans* orientation of the ligand-receptor interaction.

Some of these methodologies are then put into practice by Chang et al. who explore the molecular basis for a Siglec-7 checkpoint axis in chronic lymphocytic leukemia. In this research article the authors identify high levels of Siglec-7 ligands expressed on malignant B-cells predominately on CD43, CD45, and PSGL-1 counter receptors. The interaction of these counter receptors with Siglec-7 is facilitated through a display of disialyl-T O-glycans. This overexpression of the disialyl-T antigen likely results from overexpression of the ST6GalNAc-IV enzyme, a sialyltransferase which further sialylates the sialyl-T antigen (at the core GalNAc residue) to form the disialylated antigen. These decorations of disialyl-T antigen on malignant B-cells may inhibit anti-tumour immunity, in particular NK cell cytotoxicity, providing another example of the sialoglycan-siglec axis in tumour immunity.

Remaining of the subject of sialic acids, Villanueva-Cabelloet al. discuss and analyse the current knowledge on polysialic acid (polySia) and the immune system. Although much has been elucidated about polySia in mammals, such as its role in the central nervous system, the role of polySia in other tissues are not fully understood, including in cells of the immune system. The authors describe the dynamic changes that PolySia presents during differentiation, maturation, and activation of different types of immune cells of the innate and adaptive response. They also discuss PolySia involvement in cellular regulatory mechanisms. The paper addresses various aspects about polySia, including its biosynthesis as well as the tools for the identification and structural characterization of this glycan. Furthermore, the paper discusses various functional aspects in the immune system and its potential therapeutic implications.

Specific glycans, often on specific proteins, have been associated with cell death for decades. Parshenkov and Hennet sought to drill down into the specific pathways associated with lectin-induced cell death *via* these specific glycans. Using three lectins (Wheat Germ Agglutinin, Maackia Amurensis lectin I, Aleuria aurantia lectin) on the same cell line model, the authors demonstrated that caspase-independent but autophagy-dependent death pathways were activated. The authors conclude by arguing that the activation of these pathways may be a useful tool in sensitising tumours to other cytotoxic agents, especially those tumours that become resistant to more classically activated pathways.

Remaining of the subject of lectins, Griffiths et al. show their versatility in using them to develop a diagnostic tool for Invasive Aspergillosis (IA), a disease which is notoriously difficult to identify at an early stage. The authors examined the sequence and expression of four C-type lectin and lectin-like receptors (Dectin-1, Dectin-2, Mincle and Mcl) alongside matched responses to Aspergillus (IL6, TNF) in 42 patients. Correlation analysis revealed novel IA disease risk factors which they used to develop a pre-emptive patient stratification protocol to identify haematopoietic stem cell transplant patients at high and low risk of developing IA. 

In an analysis of patients with chronic obstructive pulmonary disease (COPD), Krick et al. identify an inverse correlation and a role of the α2,6-sialyltransferase ST6GAL1 in the production and secretion of IL-6, an important mediator of exacerbations in patients with COPD. The authors use primary patient samples and an *in vitro* system to demonstrate that low levels of ST6GAL1 increase IL-6 levels. Interestingly, they are also able to show that cigarette smoke can decrease ST6GAL1 and increase thereby IL-6.

In another disease of chronic inflammation, Wang et al. explore desialylation on synovial fibroblasts in rheumatoid arthritis (RA). Previous work had shown that RA synovial fibroblasts display lower levels of sialylation and sialyltransferases than healthy controls. Using both *in vivo* and *in vitro* models the authors explore the impact of desialylation on fibroblasts using sialidases on phenotype and function. RNA-seq analysis and protein validation showed fibroblasts became hyper-inflammatory after the removal of cell-surface sialic acids, and displayed impaired migration. The authors therefore argue that hypo-sialylation itself may be a disease driver in RA.

## Author contributions

All authors listed have made a substantial, direct, and intellectual contribution to the work and approved it for publication.

## Conflict of interest

The authors declare that the research was conducted in the absence of any commercial or financial relationships that could be construed as a potential conflict of interest.

## Publisher’s note

All claims expressed in this article are solely those of the authors and do not necessarily represent those of their affiliated organizations, or those of the publisher, the editors and the reviewers. Any product that may be evaluated in this article, or claim that may be made by its manufacturer, is not guaranteed or endorsed by the publisher.

